# Monitoring compliance with Clinical Protocol and Therapeutic Guidelines for Alzheimer’s disease

**DOI:** 10.1590/1980-57642020dn14-010004

**Published:** 2020

**Authors:** Marcela Forgerini, Patrícia de Carvalho Mastroianni

**Affiliations:** 1 PhD Student. School of Pharmaceutical Sciences, Department of Drugs and Medicines, School of Pharmaceutical Sciences, São Paulo State University (UNESP), Araraquara, SP, Brazil.; 2 Adjunct Professor at the São Paulo State University (UNESP), Department of Drugs and Medicines, School of Pharmaceutical Sciences, São Paulo State University (UNESP), Araraquara, SP, Brazil.

**Keywords:** clinical protocols, dementia, drug monitoring, drug safety, patient safety, demência, monitoramento de medicamentos, protocolos clínicos, segurança do medicamento, segurança do paciente

## Abstract

Dementia is a chronic neurodegenerative disease and Alzheimer’s disease (AD) is the most prevalent type.

**Objective:**

To describe the drug monitoring of patients enrolled in a Clinical Protocol and Therapeutic Guidelines of Alzheimer’s Disease (PCDTDA) in Brazil.

**Methods:**

A descriptive study based on interviews conducted in 2017 was performed. Patients diagnosed with Alzheimer’s disease (AD) enrolled on the PCDTDA were included. The variables assessed were age, sex, time since diagnosis, clinical parameters of Mini-Mental State Exam (MMSE) and Clinical Dementia Rating (CDR), drug therapy used and AD drug collection.

**Results:**

The drug monitoring of 143 patients was evaluated. Observing the requirements of the screening tests for patient enrolment on the PCDTDA, all patients had scores for at least one MMSE and CDR assessment at protocol admission. None of the patients underwent the first reassessment of the effectiveness of AD drug therapy or the semiannual reassessment.

**Conclusion:**

Although PCDTDA provides the best evidence of AD treatment, the data showed failures in the monitoring of the effectiveness of AD drug therapy at dispensing.

## RESUMO

A demência é uma doença crônica e neurodegenerativa, e a doença de Alzheimer (DA) é a mais prevalente.

**Objetivo:**

Descrever o monitoramento da farmacoterapia de pacientes inseridos no Protocolo Clínico e Diretrizes Terapêuticas da Doença de Alzheimer (PCDTDA), Brasil.

**Métodos:**

Estudo descritivo, conduzido por meio de entrevistas em 2017. Foram incluídos pacientes com diagnóstico da doença de Alzheimer (DA) inseridos no PCDTDA. As variáveis foram idade; sexo; tempo de diagnóstico e farmacoterapia da DA; os parâmetros clínicos Mini-exame do estado mental (MEEM) e *Clinical Dementia Rating* (CDR); e farmacoterapia em uso.

**Resultados:**

O monitoramento de 143 pacientes foi avaliado. Considerando a exigência dos testes de rastreio para a inserção do paciente no PCDTDA, observou-se que todos os pacientes tinham pelo menos um escore no MEEM e no CDR na admissão no protocolo. Nenhum paciente foi submetido à primeira reavaliação da efetividade da farmacoterapia da DA e nem à reavaliação semestral.

**Conclusão:**

Apesar do PCDTDA ser a maior evidência do tratamento da DA, dados evidenciam falhas no monitoramento da efetividade da farmacoterapia da DA na dispensação.

Dementia is a chronic neurodegenerative disease and Alzheimer’s disease (AD) is the most prevalent type.^1^ The global projection for dementia predicts 82 million cases by 2030.^2^ A meta-analysis identified Brazil as having the highest number of dementia cases^3^ among nine countries studied.

In the early stages of AD, the aim is to improve patient cognition and reduce the rate of disease progression via AD drug therapy.^4^ However, in the more advanced stages, management includes addressing the Psychological and Behavioral Symptoms of Dementia (PBSD).^4^ Palliative drug therapy is often discontinued.^5^

Drug therapy approved for the treatment of AD includes acetylcholinesterase inhibitors (donepezil, galantamine and rivastigmine) and memantine (antagonist of N-methyl-D-aspartate type glutamate receptors); these are available free of charge from the Specialized Component of Pharmaceutical Care (CEAF)^1^ according to the Clinical Protocol and Therapeutic Guidelines of Alzheimer’s disease (PCDTDA)^5^ (Table 1).

**Table 1:** Scores on MMSE and CDR screening tests for enrolment on and discharge from the Clinical Protocol and Therapeutic Guidelines for Alzheimer's Disease (PCDTDA/MS).

AD drug therapy	MMSE and CDR scores for enrolment on PCDTDA/MS	MMSE scores for discharge from the PCDTDA/MS
Anticholinesterases (donepezil, galantamine and rivastigmine)	MMSE Education > 4 years: score 12-24 Education ≤ 4 years: score 8-21 CDR: 1 and 2	MMSE Education > 4 years: score < 12 Education ≤ 4 years: score < 8
Anticholinesterase in association with memantine	MMSE Education > 4 years: score 12-19 Education ≤ 4 years: score 8-15 CDR: 2	-
Memantine monotherapy	MMSE Education > 4 years: score 5-11 Education ≤ 4 years: score 3-7 CDR: 3	MMSE Education > 4 years: score < 5 Education ≤ 4 years: score < 3

MMSE: Mini-Mental State Exam; CDR: Clinical Dementia Rating.

Diagnosing AD includes evaluating the clinical history of the patient; cognitive screening via clinical parameters such as the Mini-Mental State Exam (MMSE)^6^ and Clinical Dementia Rating (CDR);^7^ laboratory tests such as blood count, electrolytes, blood glucose, urea, creatinine, thyroid stimulating hormone, alanine aminotransferase, aspartate aminotransferase, vitamin B12, folic acid, serum serology for syphilis, and HIV in patients younger than 60 years; and magnetic resonance imaging or computed tomography.^5^

Hypothyroidism^8^ and depression^9^ are morbidities considered to be confounding factors for diagnosis^5^ because they may also cause cognitive impairment and should be investigated prior to enrolment of the patient on the PCDTDA. PBSD can also be used to evaluate previous treatment before initiating AD drug therapy.^10^

The patient should undergo a reassessment 3-4 months after enrolment onto the PCDTDA.^5^ Monitoring should be carried out every six months through clinical evaluation and MMSE and CDR screening tests to assess therapeutic effectiveness or the need for discontinuation of drug therapy due to ineffectiveness.^5^

If there is no improvement or stabilization of AD progression on the first reassessment, according to the MMSE score parameters (Table 1), then treatment should be discontinued for lack of evidence of effectiveness.^5^ This study described the monitoring of patients enrolled on the PCDTDA in the city of Araraquara, Brazil. This study was prompted by the dearth of publications on the topic and importance of compliance with the PCDTDA and monitoring of AD drug therapy.

## METHODS

This was a descriptive study involving a case series of patients diagnosed with Alzheimer’s disease enrolled on the PCDTDA and seen by the CEAF of Araraquara in 2017. Patients in long-term care institutions were excluded for ethical reasons. The study spanned one year of AD drug therapy at the CEAF.

Interviews were performed for patients, caregivers/relatives, or persons authorized to collect the AD drugs, via a standardized form. The data were confirmed via secondary sources, i.e. drug prescriptions, laboratory tests and dispensing records.

The variables of interest were age, sex, time since diagnosis, and time on AD drug therapy. The clinical parameters were MMSE and CDR scores, drug therapy used, and AD drugs collection. The variables were expressed as relative and absolute frequency.

The Research Ethics Committee of the Federal University of São Paulo (permit no. 2.877.560) approved this study. The CEAF of Araraquara declared that 260 patients were enrolled on the PCDTDA. This total comprised just one of the 17 patients included in 2017.

## RESULTS

Of the 260 patients, 49 did not collect their AD drugs, 38 individuals unaware of the treatment collected them, 16 patients were institutionalized and 14 refused to participate in the interview. Therefore, the drug monitoring of 143 patients was evaluated.

Of the patient group, most were women (67.1%), treated only under public health systems (75.5%) and practiced polypharmacy (mean of five medications/patient). Patient age ranged from 64 to 97 years and median age was 81 years (Q1=76/Q3= 87); mean time since diagnosis was four years (Q1=2/Q3=7.5).

There were 127 patients taking PCDTDA drugs in monotherapy (88.0%): 66 used galantamine (46.1%), followed by donepezil (33.6%), rivastigmine (4.9%), and memantine (4.2%). In addition, 16 patients were using anticholinesterase drugs in association with memantine (11.2%).

Use of memantine was indicated at the moderate-advanced stage of AD and identified in 22 patients. However, mean time since diagnosis of these patients was 5.5 years (SD: 2.5) based on data for only one MMSE and CDR assessment/patient (SD: 0.9).

Considering the mandatory screening tests for patient enrolment on the PCDTDA, all patients had scores for at least one MMSE and CDR assessment upon protocol admission. However, two patients did not meet the criteria for PCDTDA and should not have been receiving AD drug therapy: one patient with MMSE (26) and CDR (0) scores higher than recommended. These individuals should not have been admitted onto the protocol. Another case in use of memantine had MMSE (0) and CDR (3) scores that should have led to discharge from the PCDTDA (Table 1).

In addition, none of the patients underwent the first reassessment of the effectiveness of drug therapy AD (3-4 months) after enrolment onto the PCDTDA. Similarly, none had half-yearly reassessments. Sporadic application of the MMSE and CDR screening tests was identified in some patients, not corresponding to the monitoring recommended by the PCDTDA.

## DISCUSSION

A previous Brazilian study assessed compliance with the PCDTDA.^11^ Only one in four requests for inclusion in the PCDTDA were in accordance with the guidelines. Most requests were from patients with dementia due to Parkinson’s disease and vascular dementia (off-label use).

Hence, we hypothesize that low therapeutic impact can be expected if there is no monitoring of patients who join the PCDTDA. The results of the present study corroborate this hypothesis — there is no review of applications for inclusion of patients in the PCDTDA of Araraquara and two enrolled patients did not met the inclusion criteria.

Moreover, the absence of records of MMSE and CDR scores due to failure to-monitor the use of AD drug therapy hampered effectiveness analysis. It was not possible to confirm whether patients apparently eligible for the PCDTDA actually were, due to a lack of dispensing records. Compliant patients should have four records per year because AD drug therapy dispensing occurs every three months.

Thus, the impact of using AD drug therapy without indication or knowledge of cost-effectiveness should be explored. This is a simple economic assessment that can be performed by determining the costs of the CEAF *versus* monitored clinical parameters (MMSE and CDR). This knowledge will help explain the financial cost. However, the absence of these records precluded cost-effectiveness analysis and is a limitation of this study.

These findings corroborate the results of Picon *et al.*, who found financial waste and unnecessary patient exposure to AD drug therapy without review of patient applications for enrolment on the PCDTDA.^11^

Therefore, assessment of patient enrolment onto the PCDTDA is critical. In addition, the pharmacist-led medication therapy management (MTM) can be an effective service after the inclusion of patients on the PCDTDA, because it entails a comprehensive patient assessment considering the underlying disease (AD), confounding factors, prodromal symptoms, and therapeutic experience of the patient and caregiver/family.^12^ Previous studies on MTM involving patients diagnosed with AD resolved important problems of effectiveness^13^^,^ 14 and adherence.^15^

Another important aspect in the present study was the non-collection of AD drug therapy (49/260). This finding may be explained by the registry of patients enrolled on the PCDTDA not being up-to-date, given its size is dynamic, fluctuating with inclusions, deaths, or discharges from the PCDTDA.

An example of discharge from the PCDTDA was a patient whose swallowing issues were resolved. The antidepressant proved effective and clinical condition improved (severe depression). These improvements resulted in increased MMSE and CDR scores with consequent discharge of the subject from the PCDTDA.^13^

Several factors may be associated with this non-collection. Clearly, free AD drug therapy does not imply access. Possible barriers to this access may include limited availability of the drug, geographical accessibility, and organization of the public health service.^16^

In addition to barriers to access, some patients have access but do not adhere to the drug therapy. Thus, there are management issues of both access and the patient’s decision of whether to adhere to treatment or otherwise.

In summary, even when the patient has access to the service, is correctly diagnosed and receives AD drug therapy, these factors cannot guarantee the effectiveness of treatment. Treatment failure can be associated with the patient´s experiences and with barriers that influence safety, adherence to treatment, optimization of therapeutic results and quality of life.^17^

These findings reveal the segregation of patient care and the importance of multi-professional and integral patient assessment. This care is essential to develop health care strategies based on dementia education and care programs, because the costs of dementia in Brazil already outstrip the available resources.^18^ Therefore, strategies are required before and after enrolling patients onto the PCDTDA to ensure patient safety and sound use of AD drug therapy.
